# *Struc2mapGAN*: improving synthetic cryogenic electron microscopy density maps with generative adversarial networks

**DOI:** 10.1093/bioadv/vbaf179

**Published:** 2025-08-04

**Authors:** Chenwei Zhang, Anne Condon, Khanh Dao Duc

**Affiliations:** Department of Computer Science, University of British Columbia, Vancouver, BC V6T 1Z4, Canada; Department of Computer Science, University of British Columbia, Vancouver, BC V6T 1Z4, Canada; Department of Mathematics, University of British Columbia, Vancouver, BC V6T 1Z2, Canada

## Abstract

**Motivation:**

Generating synthetic cryogenic electron microscopy 3D density maps from molecular structures has potential important applications in structural biology. Yet existing simulation-based methods cannot mimic all the complex features present in experimental maps, such as secondary structure elements. As an alternative, we propose *struc2mapGAN.*

**Results:**

*Struc2mapGAN* is a novel data-driven method that employs a generative adversarial network to produce improved experimental-like density maps from molecular structures. More specifically, *struc2mapGAN* uses a nested U-Net architecture as the generator, with an additional L1 loss term and further processing of raw training experimental maps to enhance learning efficiency. While *struc2mapGAN* can promptly generate maps after training, we demonstrate that it outperforms existing simulation-based methods for a wide array of tested maps and across various evaluation metrics.

**Availability and implementation:**

The* struc2mapGAN* is publicly accessible via https://github.com/chenwei-zhang/struc2mapGAN.

## Introduction

Cryogenic electron microscopy (cryo-EM) is a powerful technique for structural determination of biological macromolecules such as proteins and nucleic acids, which has been widely used in structure-based drug discovery (Merk *et al.* 2016, Renaud *et al.* 2018). Cryo-EM produces a series of 2D images that are then reconstructed into electron density maps, providing a 3D voxelized representation of the macromolecular complex.

For the past few years, deep learning-based methods have been introduced to automate the construction of macromolecular atomic models from density maps (i.e. 3D coordinate structure) (Pfab *et al.* 2021, He *et al.* 2022, Jamali *et al.* 2024). However, most of these methods require high-fidelity experimental maps, given the lack of reliable experimentally derived map-model pairs at lower resolutions (Wriggers and He 2015). In this context, generating accurate synthetic maps can help to include the vast majority of structures that are limited to being solved at lower resolutions. In addition, these can also be used for “rigid-body fitting” in the building process (Tajmir Riahi *et al.* 2025), “sharpening” density maps (i.e. enhancing the map to facilitate model building) (He *et al.* 2022), or providing more ground-truth training targets (Dai *et al.* 2023, He *et al.* 2023, Maddhuri Venkata Subramaniya *et al.* 2023).

As generating synthetic cryo-EM density maps has become a pivot, various simulation-based methods are available (Ludtke *et al.* 1999, Pettersen *et al.* 2004, Tang *et al.* 2007, Wriggers 2012, Cragnolini *et al.* 2021), essentially using a resolution-lowering point spread function, as detailed in the next section, to convolute atom points extracted from PDB structures (atomic models). Upon treating atoms independently, these methods may fail to characterize more complex features, such as secondary structure elements (SSEs), interatomic interactions, or specific image artifacts inherent to the 3D reconstruction process. As a result, notable discrepancies can be observed between simulated maps generated by tools like *molmap* in ChimeraX (Pettersen *et al.* 2004) and actual experimental maps, particularly in the representation of SSEs and local density patterns. To address this gap, a potential solution is to leverage a deep generative model to learn a mapping from simulated to experimental maps. This led us to develop and present here a new method, called *struc2mapGAN*, that uses a generative adversarial network (GAN) to enhance the generation of simulations of cryo-EM density maps from PDB structures and current simulation methods. Our contributions are as follows.

- We introduce the first deep learning-based method, to our knowledge, for generative modeling of cryo-EM density maps. Our method enhances the learning efficiency by curating high-quality training data and alleviates mode collapse (Zhang *et al.* 2018) in GANs through the integration of SmoothL1Loss into the model.
- Our benchmarking shows superior overall performance against simulation-based methods across various evaluation metrics (e.g. correlation) and over a variety of tested maps. Our experiments also suggest that this performance improvement results from better capture of SSEs.
- We benchmark the runtime of *struc2mapGAN* and demonstrate its practical suitability for generating large-scale maps.

## Related work

### Existing simulation-based methods

Simulation-based methods for generating density maps from their associated PDB structures are based on the convolution of atom points with resolution-lowering point spread functions such as Gaussian, triangular, or hard-sphere (Bracewell and Kahn 1966, Gonzalez and Woods 2009). Given a PDB structure containing *M* atoms, a general Gaussian simulation formula for producing a density value ρ at a grid point x is expressed as:


(1) $$\rho (x)=\sum_{i=1}^{M}\theta Z_{i}e^{-k|x-r_{i}{|}^{2}},$$

where $Z_{i}$ represents the atomic number and $r_{i}$ is the position vector of the *i*-th heavy atom, θ is a scaling factor, and *k* is defined in terms of the resolution (He *et al.* 2023, 2022). Such methods including *e2pdb2mrc* in EMAN2 (Tang *et al.* 2007) [originally called *pdb2mrc* in EMAN1 (Ludtke *et al.* 1999)], *molmap* in UCSF ChimeraX (Pettersen *et al.* 2004), *StructureBlurrer* in TEMPy2 (Cragnolini *et al.* 2021), and *pdb2vol* in Situs (Wriggers 2012), generate 3D density maps using a Gaussian point spread function, where the real-space dimension corresponds to the desired resolution, which varies based on the specific resolution convention of each package.

### Generative adversarial networks

GAN is one of the most prevalent generative models widely employed in image generation, super-resolution, and 3D object generation (Creswell *et al.* 2018). The GAN architecture comprises two main models, a generator *G* and a discriminator *D* trained together. *G* generates a batch of images (we call them fake images), and these images are fed along with real images (ground-truth reference images) into *D* to be classified as real or fake. During training, *G* generates fake images to fool *D*, while *D* is updating its parameters to discriminate the fake ones. Mathematically speaking, the two models, *G* and *D*, compete in a two-player minimax game with the objective function L(G,D):


(2) $$\underset{G}{\min}\underset{D}{\max}V(D,G)=E_{x}[\text{log}\;D(x)]+E_{z}[\text{log}(1-D(G(z)))].$$

The generator minimizes the log(1−D(G(z))) term predicted by *D* for fake images. Conversely, the discriminator maximizes the log probability of real images, log D(x) and the log probability of correctly identifying fake images, log(1−D(G(z))). Over the past few years, the GAN architecture has been adapted for various purposes and has demonstrated superior performance in multiple domains (Isola *et al.* 2017, Zhu *et al.* 2017, Adler and Lunz 2018, Gupta *et al.* 2021, Maddhuri Venkata Subramaniya *et al.* 2023).

## Methods

### Datasets

#### Training and validation data

To train and validate the GAN model, our dataset was built with a set of high-resolution cryo-EM density maps ranging from 2.2 Å to 3.9 Å from the EMDB databank (Lawson *et al.* 2016) and associated PDB structures from the PDB databank (Berman *et al.* 2002). To ensure that the density maps have proper alignments with their associated PDB structures, we removed maps from the dataset if: (i) maps contain extensive regions without corresponding PDB structures; (ii) maps are misaligned with associated PDB structures; (iii) maps contain various macromolecules, such as nucleic acids; (iv) PDB structures only contain backbone atoms and/or include unknown residues. In addition, pairs of simulated and experimental maps with correlation lower than 0.65 (calculated using ChimeraX) were excluded. To eliminate data redundancy, we measured the sequence identity between PDB structures and retained only one when the identity exceeded 30%. After applying these filtering steps, a total of 149 cryo-EM density maps and associated PDB structures remained, as listed in Supplementary Table S1. 134 map-PDB pairs (90%) were selected as the training set and 15 pairs (10%) as the validation set.

#### Test data

For the test set, we randomly selected 130 PDB structures from the PDB databank and associated cryo-EM density maps from the EMDB databank with resolution ranging from 3 Å to 7.9 Å, as detailed in Supplementary Table S2. Note that these 130 examples do not overlap with the training and validation data sets.

#### Preprocessing

**Figure 1.**
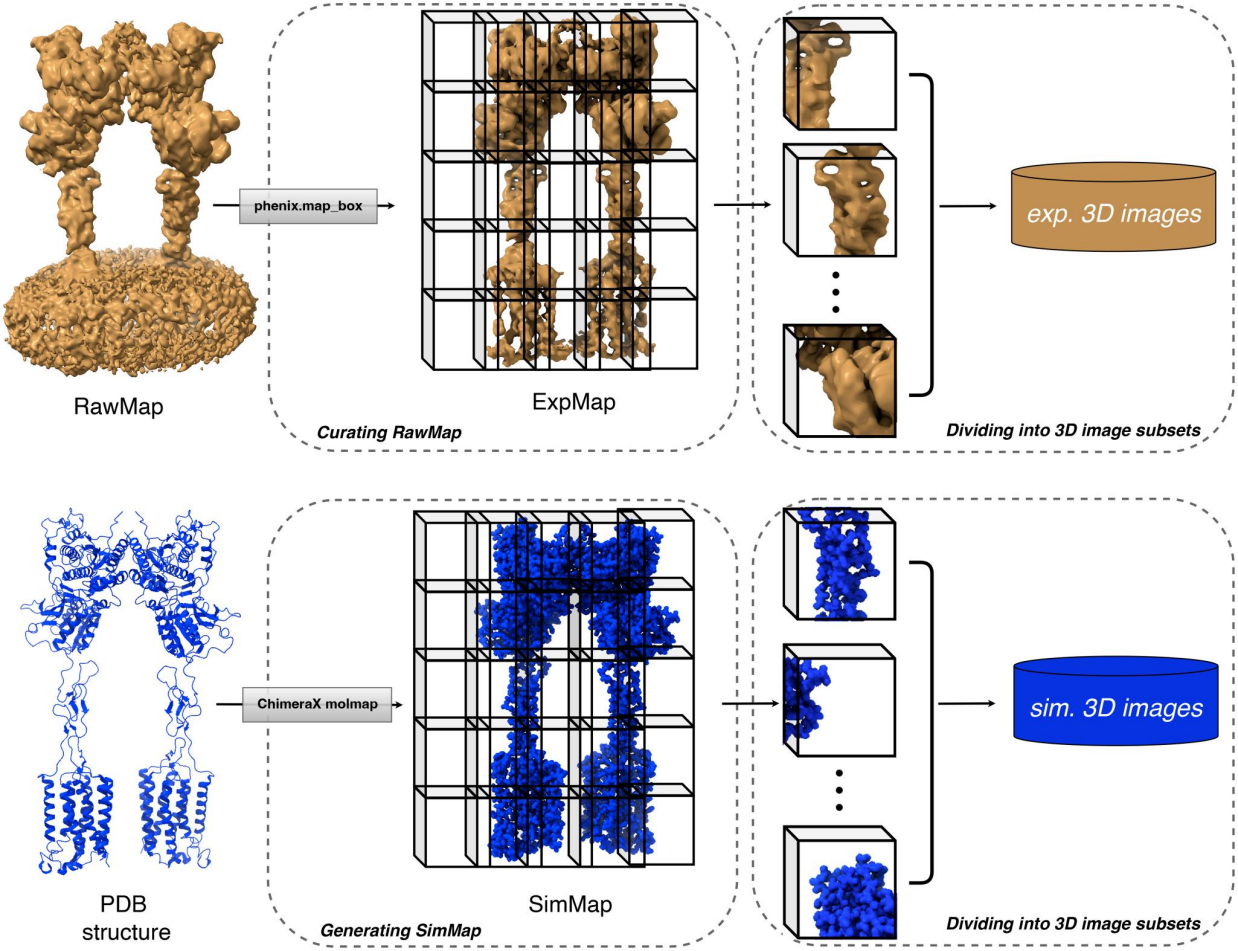
The data preprocessing workflow. The top panel depicts the process of curating raw experimental maps and dividing the curated maps into 3D image subsets as training targets. The bottom panel depicts the process of generating simulated maps and dividing them into 3D image subsets as training inputs. The illustrated structure is Metabotropic Glutamate Receptor 5 Apo Form (EMDB ID: 0346; PDB ID: 6N52; reported resolution: 4 Å) (Koehl *et al.* 2019).

According to the following preprocessing steps illustrated in Fig. 1, both experimental maps (denoted as RawMaps) and simulated maps from PDB structures (denoted as SimMaps) were preprocessed to facilitate the GAN training. In addition, our GAN architecture also takes 3D images of reduced size as input.

#### Curating RawMaps

The raw experimental maps consistently contain background noise, such as lipid solvents and artifacts from nanodiscs, which compromise the pairing accuracy between RawMaps and SimMaps. To mitigate these effects, we applied a mask to the raw map to isolate the region containing only the protein structure. We first aligned the PDB structure with the corresponding map and employed *phenix.map_box* to create a rectangular box slightly larger than the targeted region. Subsequently, we resampled the masked map at a grid voxel size of 1Å×1Å×1Å and applied min–max normalization to scale the map’s voxel values to the range of [0, 1] to maintain uniformity. The curated map, termed ExpMap, was then employed for training the network. Note that this curation strategy to make ExpMap targets significantly enhanced the performance of the GAN model, as evidenced in our ablation study section.

#### Generating *SimMaps*

The input data SimMaps were simulated using the *molmap* function. This step provides clean and standardized inputs, allowing the network to learn the mapping toward more realistic experimental maps. We converted the PDB structure into a simulated density map on a grid corresponding to the ExpMap, with a resolution cutoff at 2 Å. This simulated map was then min–max normalized to the range of [0, 1], aligning with its corresponding ExpMap. Moreover, in light of enhancing the model’s robustness, we utilized *TorchIO* (Pérez-García *et al.* 2021) to add random Gaussian noise, random anisotropy, and random blur to augment the input data.

#### Dividing maps into 3D image subsets

Considering the varying dimensions of each map and the constraints of GPU memory, we zero-padded the SimMaps and ExpMaps and divided them into smaller 3D images (denoted as *exp. 3D images* and *sim. 3D images*, respectively) with dimensions of 32×32×32. To do so, we first created a padded map that exceeded the dimensions of the input map by 2×32 in each dimension, ensuring no boundary issues. The original map was then centrally placed within this padded map. Following this procedure yielded a total of 403 710 3D images for training and 62 635 for validation.

### The *struc2mapGAN* architecture

**Figure 2.**
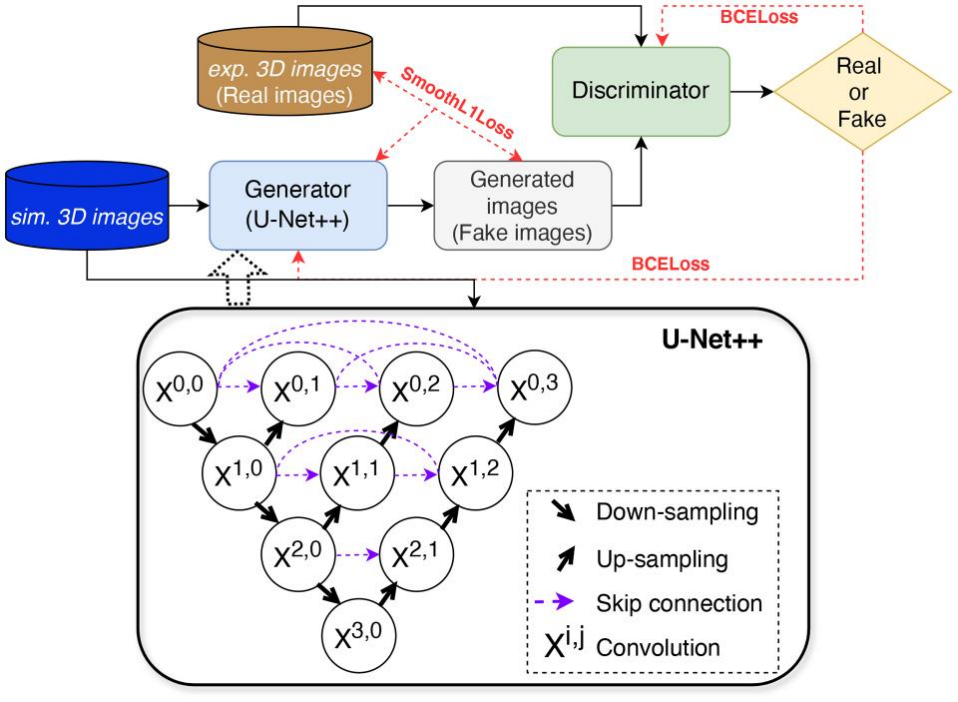
The *struc2mapGAN* architecture. The bottom panel illustrates the U-Net++ architecture. $X^{i,j}$ refers to the convolution block at depth *i* and position *j* of the network.

Figure 2 depicts the *struc2mapGAN* architecture that comprises a generator and a discriminator. We have implemented a nested U-Net architecture (U-Net++) (Zhou *et al.* 2018) as the generator (shown in the bottom panel of Fig. 2). The encoder and decoder blocks of the U-Net++ follow the same design, each consisting of 3D convolution layers with a kernel size of 3×3×3. Unlike standard U-Nets, U-Net++ applies dense skip connections that effectively bridge the encoder and decoder feature maps for enhanced gradient flow. 3D max-pooling layers, featuring a kernel size of 2 and a stride of 2, are employed for down-sampling, while trilinear interpolation layers with a scale factor of 2 are used for up-sampling.

The discriminator network has been designed for 3D volumetric data classification. It consists of four 3D convolution layers with a kernel size of 3×3×3. An adaptive average-pooling layer is applied after the last convolution layer to reduce the feature map to the size of 1×1×1, which is then flattened and passed through three fully connected layers with ReLU activations between them. The final layer outputs a single value for binary classification.

For both the generator and discriminator, we employed a parametric rectified linear unit (PReLu) as the activation function between convolution layers, coupled with instance normalization. We opted for instance normalization over the more commonly used batch normalization, as the statistics should not be averaged across instances within a batch. This consideration is crucial since local 3D image subsets of cryo-EM density may originate from different maps. The total number of parameters for the generator is 10.3 million, and for the discriminator, it is 1.2 million.

### Training and inference of *struc2mapGAN*

#### Training

During training, *struc2mapGAN* accepts paired *sim. 3D images* and *exp. 3D images* as input and outputs modified images. To impose the generator to minimize the difference between the prediction and the target, we incorporated the standard adversarial loss for each input *sim. 3D image* with the smooth L1 loss in the generator, as


(3) $$\text{SmoothL}1\text{Loss}(X,Y)=\{\begin{array}{cc} 0.5{\left(X-Y\right)}^{2}, & \text{if}\;|X-Y|<1,\\ |X-Y|-0.5, & \text{otherwise}, \end{array}$$

where *X* is the GAN-predicted image, i.e. G(sim.3Dimage), and *Y* is the paired target image, i.e. *exp. 3D image*. The adversarial loss is calculated as the binary cross entropy loss of the discriminator’s predictions, as


(4) $${\text{Loss}}_{\text{adversarial}}=-\text{log}(D(X)).$$

Therefore, the generator loss is formulated as a linear combination of adversarial loss and smooth L1 loss:


(5) $${\text{Loss}}_{G}=\text{SmoothL}1\text{Loss}+\alpha \times {\text{Loss}}_{\text{adversarial}},$$

where α is a tuning hyperparameter. The discriminator loss adheres to the standard GAN loss formula, aiming to classify *X* as fake and *Y* as real:


(6) $${\text{Loss}}_{D}=-(\text{log}\;D(Y)+\text{log}(1-D(X))).$$

#### Inference

To generate a map from a PDB structure using our trained generator, the input PDB structure was initially converted to a map following the procedure of generating SimMaps. This converted map was subsequently zero-padded and divided into 3D image subsets with dimensions of 32×32×32, following the same strategy employed during training data preprocessing. These image subsets were then refined by the trained generator to yield post-processed images. The post-processed images were then reassembled back to their original map dimensions. To ensure that no spatial information was lost, we exclusively utilized the center 20×20×20 voxels of each 3D image to reconstruct the map, in accordance with the method proposed by Si et al. (Si *et al.* 2020).

#### Implementation

*Struc2mapGAN* was implemented in PyTorch-2.2.2 + cuda-12.1, with all training and validating processes carried out on eight NVIDIA A100 GPUs, each with 80 GB VRAM. This setup supported a batch size of 128. The network was trained over 150 epochs, with each epoch taking approximately 765 seconds. The total training duration was approximately 1 day and 8 hours. For optimization, NAdam (Dozat, 2016) optimizers were employed with a learning rate set at 0.0001. We tested three different α values: 0.1, 0.01, 0.001. Ultimately, α=0.01 was selected for yielding the best performance.

### Evaluation metrics

To measure the accuracy of *struc2mapGAN* in generating maps that are similar to experimental reference ones, we used the following evaluation metrics.

#### Structural similarity index measure

The structural similarity index measure (SSIM) evaluates the similarity between two images based on three key features: luminance, contrast, and structure (Wang *et al.* 2004). This measure has been extended to evaluate 3D volumetric data and has been adapted as a loss function in cryo-EM density map studies (He *et al.* 2023, 2022). The SSIM score between samples *X* and *Y* is calculated as follows:

(7) $$\text{SSIM}(X,Y)=\frac{\left(2\mu_{X}\mu_{Y}+c_{1})(2\sigma_{XY}+c_{2}\right)}{\left(\mu_{X}^{2}+\mu_{Y}^{2}+c_{1})(\sigma_{X}^{2}+\sigma_{Y}^{2}+c_{2}\right)},$$

where $\mu_{X}$ and $\mu_{Y}$, $\sigma_{X}$ and $\sigma_{Y}$ are the means and variances of samples *X* and *Y*, respectively. $\sigma_{X}Y$ refers to the covariance of *X* and *Y*. Constants $c_{1}$ and $c_{2}$ are included to stabilize the division. SSIM excels in capturing the local contrast and textures, which are crucial in cryo-EM density maps. In our study, we employed the *scikit-image* package to calculate the SSIM between a *struc2mapGAN*-generated or simulation-based density map and its corresponding experimental map.

#### ChimeraX correlation

UCSF ChimeraX (Pettersen *et al.* 2004) provides a built-in *measure correlation* function to calculate the correlation between two density maps in two ways. One way is to compute the correlation between two maps directly without considering the deviations from their means:

(8) $$\text{correlation}(X,Y)=\frac{\langle X,Y\rangle }{∥X∥∥Y∥},$$

where 〈X,Y〉 represents the dot product of maps *X* and *Y*; ∥X∥∥Y∥ are the norms of the maps. The other way calculates the correlation between deviations of two maps from their means:

(9) $$\text{correlation}\;\text{about}\;\text{mean}(X,Y)=\frac{\langle X-X_{\text{ave}},Y-Y_{\text{ave}}\rangle }{∥X-X_{\text{ave}}∥∥Y-Y_{\text{ave}}∥},$$

where $X_{\text{ave}}$ and $Y_{\text{ave}}$ are the means of the two maps. In the context of comparing cryo-EM density maps, we calculated the two correlations to assess the similarity across the entire volume of the two maps.

#### Pearson correlation coefficient

The Pearson correlation coefficient (PCC) is a measure of the linear correlation between two datasets (Sedgwick 2012) and is defined by:

(10) $$\text{PCC}(X,Y)=\frac{\sum_{i=1}^{n}\left(X_{i}-\bar{X}\right)(Y_{i}-\bar{Y})}{\sqrt{\sum_{i=1}^{n}{\left(X_{i}-\bar{X}\right)}^{2}\sum_{i=1}^{n}{\left(Y_{i}-\bar{Y}\right)}^{2}}},$$

where $X_{i}$ and $Y_{i}$ represent the individual data points in datasets X and *Y*, respectively. $\bar{X}$ and $\bar{Y}$ are the means of the datasets, and *n* is the total number of data points. In the context of comparing cryo-EM density maps, the PCC provides similarities between the two maps in terms of their density distributions.

## Results

We conducted a comprehensive study to evaluate the performance of *struc2mapGAN* using a test set of 130 PDB structures and associated experimental density maps (note that these structures and maps were not used for training the GAN network). In our benchmarking, simulation-based maps were generated using various methods (*molmap*, *e2pbd2mrc*, and *StructureBlurrer*), with a resolution cutoff at 2 Å.

### Learning curves with intermediate maps

**Figure 3.**
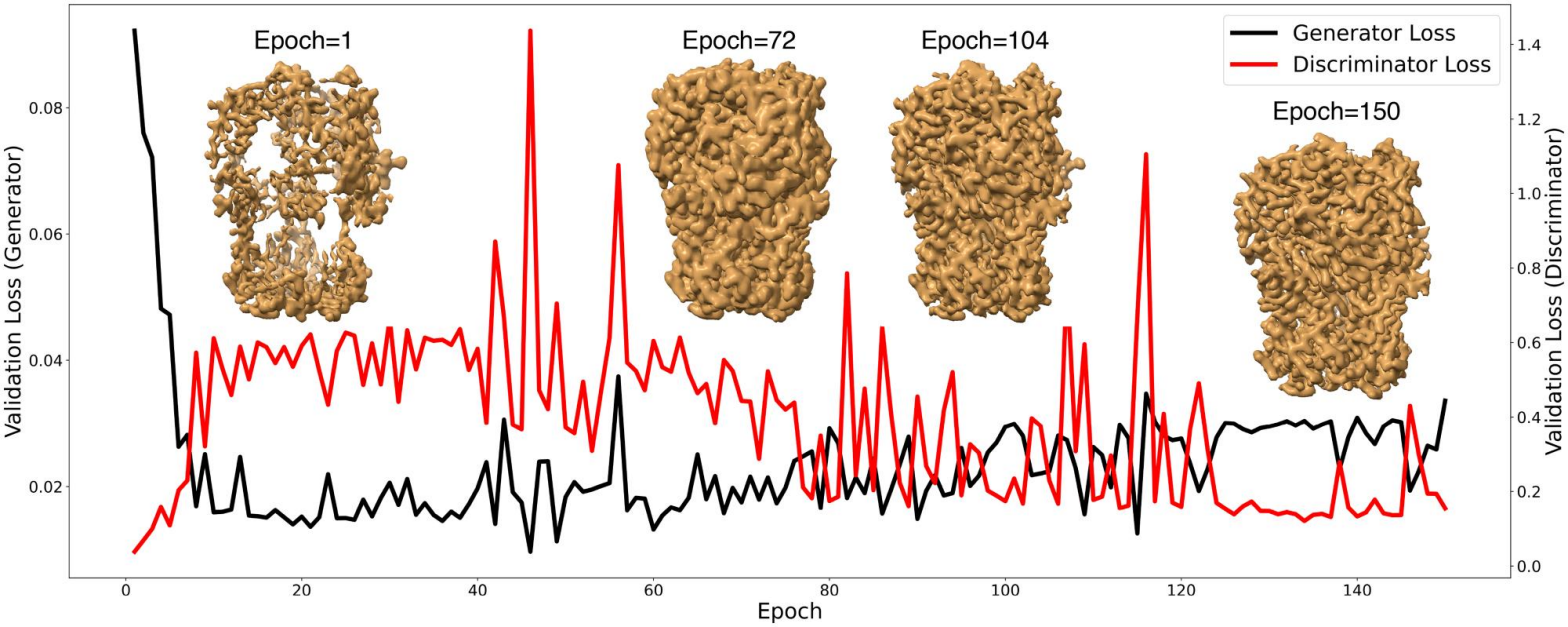
Validation loss curves of the generator and discriminator in black and red, respectively, with snapshots of generated maps from the models trained at specific epochs. The illustrated structure is Rotavirus VP6 (EMDB ID: 6272; PDB ID: 3J9S; reported resolution: 2.6 Å (Grant and Grigorieff 2015)).

To analyze the training process, we visualized the training dynamics of *struc2mapGAN* in Fig. 3. The validation losses of both the generator (black) and discriminator (red) were plotted over 150 epochs. At the beginning of training, the generator loss decreased sharply, reflecting its quick adaptation to produce more realistic maps, while the discriminator loss slightly increased owing to the generator’s enhancements. Throughout the training, fluctuations in both losses indicate continuous learning and adaptation. By the end of the training, the losses of both the generator and discriminator tended to converge, suggesting a balanced and stabilized adversarial training outcome. Alongside the loss curves, selected *struc2mapGAN*-generated maps from epochs 1, 72, 104, and 150 were displayed. These intermediate maps visually encapsulated the gradual improvement in the quality and fidelity of the generated maps. For instance, the map from Epoch 1 showed incomplete densities. As training proceeded, the generated maps learned to represent complete densities, with their resolution progressively refined, as illustrated in the maps from Epoch 72 to Epoch 150.

### Visual comparison between generated maps by *struc2mapGAN* and molmap

**Figure 4.**
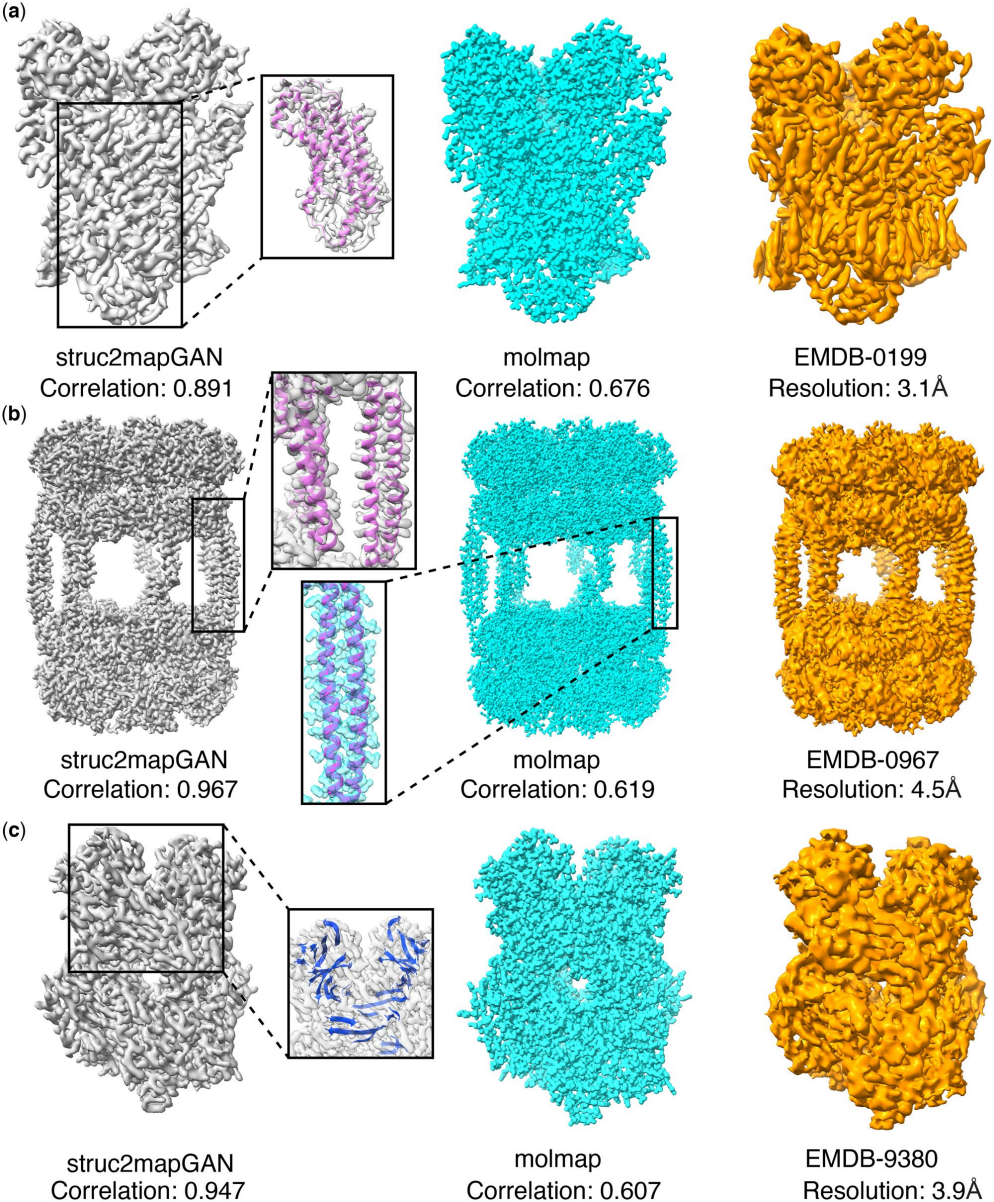
Examples of *struc2mapGAN* (gray) and *molmap* (cyan) generated maps, and the raw experimental maps (orange). The PDB structures of α-helices (pink) and β-sheets (blue) are superimposed on the maps. (a) Human STEAP4 bound to NADP, FAD, heme and Fe(III)-NTA (EMDB ID: 0199; PDB ID: 6HCY; reported resolution: 3.1 Å) (Oosterheert *et al.* 2018). (b) AAA+ ATPase, ClpL from Streptococcus pneumoniae: ATPrS-bound (EMDB ID: 0967; PDB ID: 6LT4; reported resolution: 4.5 Å) (Kim *et al.* 2020). (c) Truncated HIV-1 Vif/CBFbeta/A3F complex (EMDB ID: 9380; PDB ID: 6NIL; reported resolution: 3.9 Å) (Hu *et al.* 2019). Visualization of cryo-EM density maps and PDB structures was produced by UCSF ChimeraX (Pettersen *et al.* 2004).

Upon training, we first visualized three GAN-generated density maps alongside their associated *molmap*-generated maps, with corresponding experimental maps ranging from high to low resolutions. The visual comparisons are displayed in Fig. 4. We observed that as *struc2mapGAN* is trained to represent high-resolution maps, the GAN-generated maps were produced with a consistent detail level across the three examples, with high correlation scores (0.891, 0.967, and 0.947) with experimental maps. In contrast, *molmap*-simulated maps displayed fine-grained resolution at the level of individual atoms (0.676, 0.619, and 0.607). In practice, these maps effectively captured the spatial distribution of atomic densities but did not seem to distinctly represent SSEs (see on-set panels in Fig. 4). In contrast, *struc2mapGAN* better captures SSEs such as α-helices and β-sheets, as GAN-generated maps for EMDB-0967 and EMDB-9380 distinctly revealed in Fig. 4b and c the densities of helical and sheet structures, respectively, indicating that our model effectively learned the complexity of experimental maps. We further examined whether similar visual characteristics could be replicated by adjusting the resolution and threshold parameters in *molmap*. As shown in Supplementary Figure S1, tuning these parameters primarily smooths the map surface but does not recover SSEs such as β-sheets, which remain absent in the *molmap*-simulated maps. In contrast, *struc2mapGAN*-generated maps distinctly reveal such features. This highlights that *struc2mapGAN* goes beyond surface-level smoothing by learning to enhance biologically meaningful structural elements that simulation-based methods fail to reproduce. These visual results demonstrate that the maps synthesized by *struc2mapGAN* achieved high visual resolution quality, with improved representation of SSEs.

### Quantitative comparison over resolution

**Figure 5.**
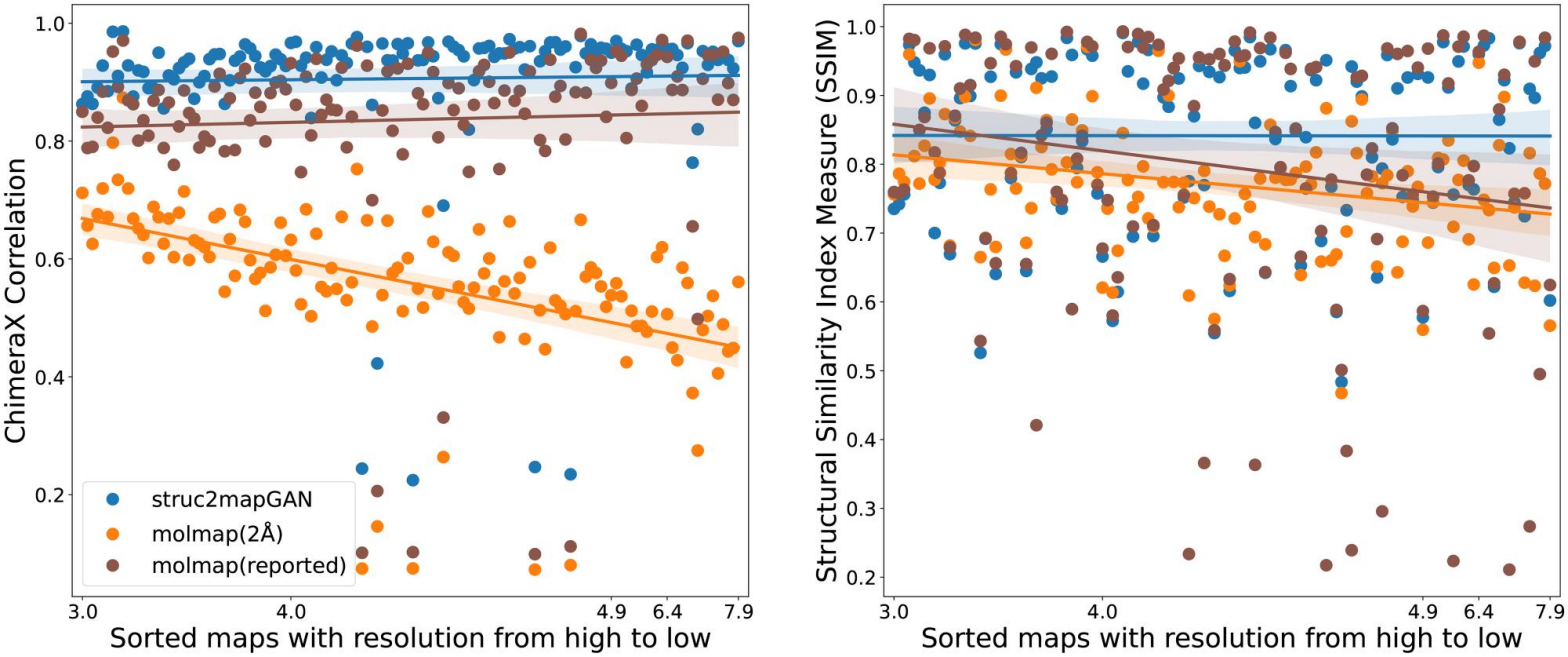
The scatter plots for comparison of ChimeraX correlation (left) and SSIM (right) for *struc2mapGAN* (blue dots), *molmap* at a resolution cutoff at 2 Å (orange dots), and *molmap* at the reported resolution cutoff (brown dots), across 130 test examples. Each point represents one test sample, and the X-axis reflects the sorted order of maps based on their reported resolution, from high (left) to low (right). The x-tick values correspond to representative resolution values (in Å) sampled from the dataset. Because many maps share identical resolution values, these tick marks are not evenly spaced along the axis. The shaded area around each colored regression line represents the confidence interval of the regression estimate.

We then studied how the GAN-generated maps from PDB structures compare against the corresponding experimental counterpart and how a simulation-based method like *molmap* performs in comparison. For this section, we also aimed to consider the resolution of the experimental map [ranging from high (3 Å) to low (7.9 Å)], since GAN-generated maps aim to replicate the map features at high resolution. As depicted in Fig. 5, over 95% of GAN-generated maps achieved high correlation scores above 0.8, irrespective of their resolution. Surprisingly, this correlation was also constant on average across resolution levels (as shown by the fitted curve). A similar result was obtained using SSIM (see Methods). We hypothesize that the information loss in low-resolution maps, which would result in poor placement of atoms in the PDB structure, was compensated by incorporating high-resolution training data, leading to the maintenance of performance in low-resolution maps. To support this hypothesis, we performed the same comparison using *molmap*-simulated maps at both a high resolution of 2 Å, and the same reported resolution as the experimental one. In Table 1, we report the mean and median correlation and SSIM scores that indicate better performance of GAN-generated maps. As expected, simulated maps at 2 Å showed poorer correlation and similarity as resolution decreases, presumably due to a mismatch between the fine atomic features emphasized in high-resolution simulated maps and the blurred, noisy nature of corresponding experimental maps—particularly at lower resolutions (see Fig. 5). This mismatch reduces voxel-wise correlation despite the apparent precision of simulated maps. Simulated maps at reported resolution would maintain constant correlation (compensating atom misplacement by a more diffuse kernel), but with less value than *struc2mapGAN*, and also a decreasing trend for SSIM, supporting that GAN-generated maps can compensate for the uncertainty of atomic positions in PDB structures generated at low resolution.

**Table 1.** Performance comparison of different methods.

	SSIM		Correlation		Correlation about mean		PCC	
Methods	Mean	Median	Mean	Median	Mean	Median	Mean	Median
***struc2mapGAN***	0.841	**0.896**	**0.906**	**0.943**	**0.466**	**0.502**	**0.594**	**0.621**
Molmap	0.771	0.774	0.559	0.573	0.315	0.311	0.452	0.470
StructureBlurrer	0.764	0.771	0.603	0.620	0.335	0.334	0.475	0.499
e2pdb2mrc	**0.848**	**0.896**	0.613	0.649	0.322	0.325	0.483	0.519

Bold values indicate the best-performing scores.

### Benchmarking

**Figure 6.**
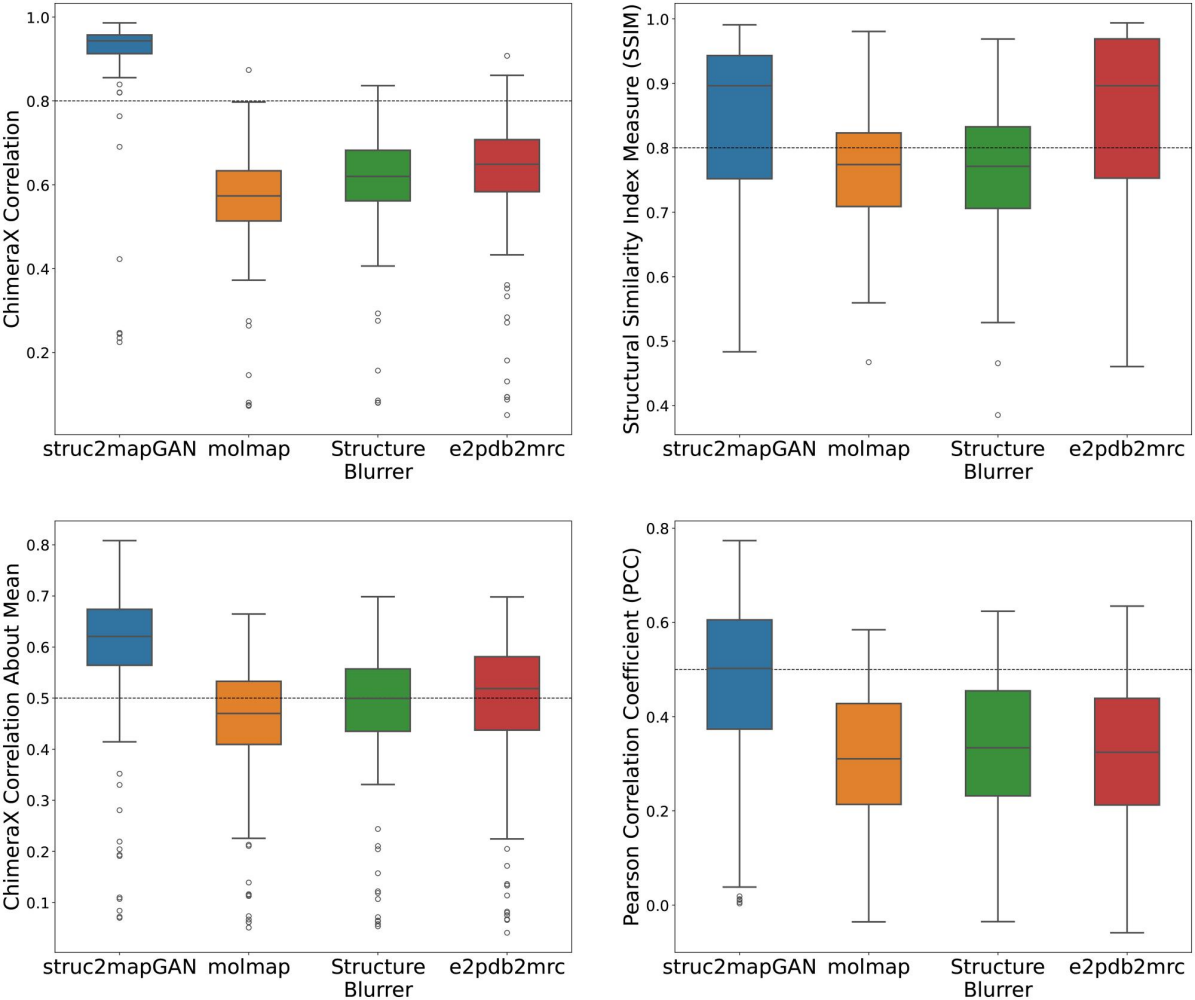
The box-whisker plots for comparison of different methods (*struc2mapGAN*, *molmap*, *StructureBlurrer*, and *e2pdb2map*) across four evaluation metrics over 130 test examples. For each box-whisker plot, the center line is the median; lower and upper hinges represent the first and third quartiles; the whiskers stretch to 1.5 times the interquartile range from the corresponding hinge; and the outliers are plotted as circles.

We further compared *struc2mapGAN* with other commonly used simulation-based methods, including *StructureBlurrer* and *e2pdb2mrc*, in terms of SSIM, correlation, and PCC scores across all 130 test examples. The mean and median values of these metrics are listed in Table 1. According to the box-and-whisker plots shown in Fig. 6, maps generated by *struc2mapGAN* consistently produced higher scores in metrics of correlation, correlation about mean, and PCC compared to the other methods. Specifically, *struc2mapGAN* achieved an average correlation of 0.906 and a PCC of 0.594, significantly surpassing the other methods. Although *struc2mapGAN*’s average SSIM score was 0.841, slightly lower than *e2pdb2mrc*’s 0.848, it exhibited a narrower interquartile range, indicating more consistent results. The high SSIM scores achieved by *e2pdb2mrc* can be attributed to its effective preservation of local structural details. This preservation is facilitated by the method’s requirement to select a larger box size (500Å×500Å×500Å) than the reference for successfully generating a density map. A larger box size encompasses more spatial context around the molecule, thereby retaining more local structural details. Aside from the SSIM score for *e2pdb2mrc*, we also observe that all three simulation-based methods yield comparable results across all metrics, showing their relative similarity.

### Map generation time

**Figure 7.**
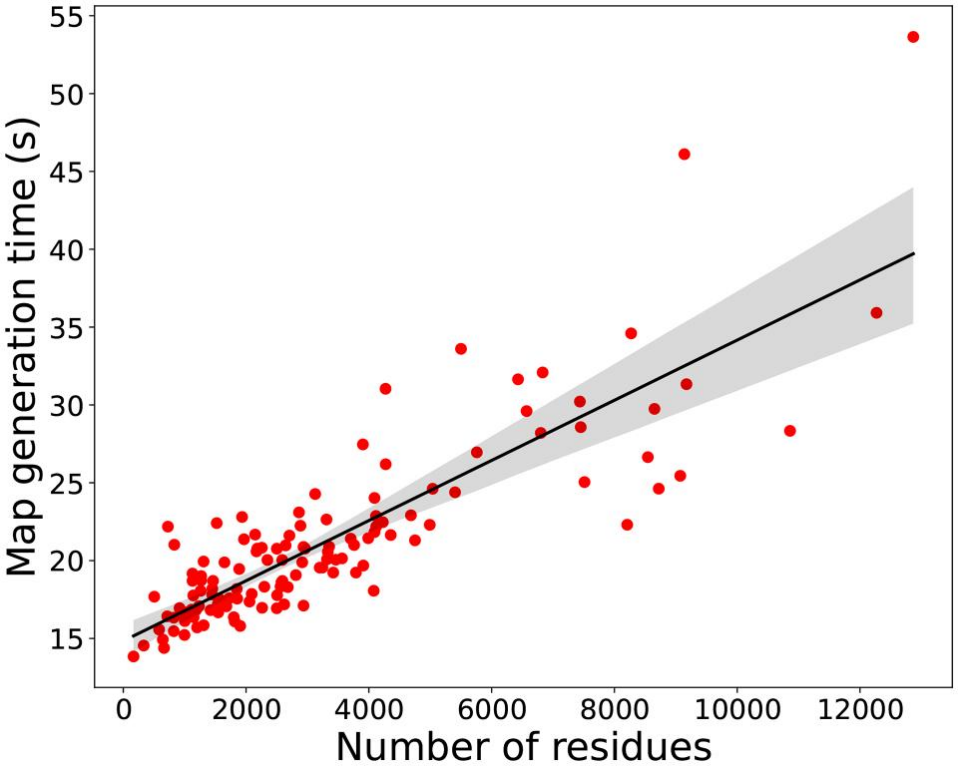
The scatter plot of map generation time against the number of residues in the molecule. Each red dot represents a molecule’s generation based on its residue count. The shaded area around the black regression line represents the confidence interval of the regression estimate. Running times were recorded for an AMD Ryzen Threadripper 2950X Processor with 32 CPUs.

To assess the potential use of *struc2mapGAN* in practice, we also recorded the time to generate experimental-like density maps from PDB structures for all 130 test examples. Figure 7 shows the wall-clock time plotted against the number of residues of each candidate structure. The scatter plot indicates that the relationship between the map generation time and the number of residues is approximately linear. Upon reviewing several instances, we found that it took around 14 and 20 seconds to generate maps containing 644 and 2501 residues, respectively. For a significantly larger structure with 12868 residues, the map generation time remained within an acceptable range, approximately 53 seconds. These results demonstrate that *struc2mapGAN* scales efficiently with the increasing complexity of protein structures, making it a viable tool for real-time applications.

### Ablation study

**Figure 8.**
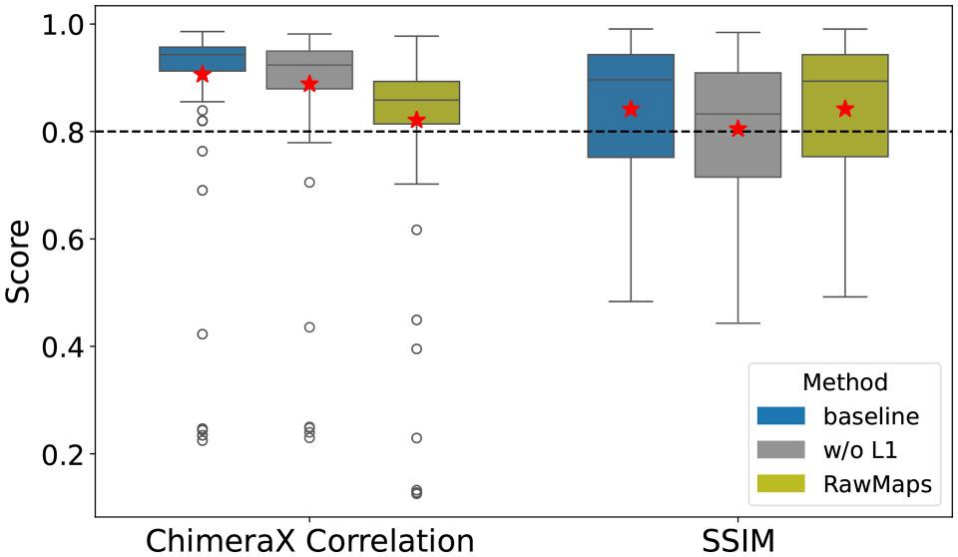
The box-whisker plots for comparison among *struc2mapGAN* (base), the network trained without SmoothL1Loss (w/o L1), and the network trained with uncurated raw experimental maps (RawMaps). The evaluation was made across two metrics: ChimeraX correlation and SSIM, based on 130 test examples. The red stars represent the mean values of corresponding scores.

Two key factors influence the learning efficiency of *struc2mapGAN*. The first is the incorporation of SmoothL1Loss in the generator as an additional constraint to mitigate mode collapse inherent in GANs and stabilize the training process. The second is the use of curated experimental maps as the learning targets, enabling the model to learn more accurate and reliable mappings between synthetic and experimental maps. To investigate the impact of these factors on the performance of *struc2mapGAN*, we trained two distinct GAN models: one without SmoothL1Loss (w/o L1) and the other using raw experimental maps without any curation. We then conducted these evaluations on both models using all 130 test examples. ChimeraX correlation scores are reported in Fig. 8. The baseline *struc2mapGAN* (i.e. trained with SmoothL1Loss and using curated maps) achieved the highest mean and median scores, as well as the narrowest interquartile range. These results indicate that incorporating SmoothL1Loss enhances the preservation of overall similarity in the generated maps and leads to more consistent outcomes. Similarly, SSIM scores are reported in Fig. 8, indicating slightly lower mean and median without SmoothL1Loss.

The model trained with only raw maps achieved SSIM scores with a mean of 0.842 and a median of 0.894, comparable to those of *struc2mapGAN* (mean of 0.841 and median of 0.896). This result was anticipated since the SSIM metric is more sensitive to local structural details. Training with raw maps maintained the local molecular information, resulting in minimal variance in SSIM scores. However, most noise, artifacts, and solvents were removed when raw maps were curated, which allows *struc2mapGAN* trained with these curated maps to focus more on overall similarity, thereby yielding higher correlation scores. These findings underscore the importance of using an additional SmoothL1Loss to guide network training and employing curated maps as input targets, both of which enhance the model performance.

## Conclusion and discussion

In this work, we present *struc2mapGAN*, a novel method adapted from the GAN architecture to generate cryo-EM density maps from PDB structures, which mimic the unique characteristics of raw maps. We enhance *struc2mapGAN*’s training efficiency by excluding the noise and artifacts and selectively extracting the macromolecule regions from the raw maps as training input, as well as incorporating SmoothL1Loss into the generator. Our benchmarking results show that *struc2mapGAN* outperforms existing simulation-based methods across various evaluation metrics. Moreover, its rapid synthesis speed makes it suitable for generating large-scale data.

Recent works have made significant progress in simulating realistic 2D cryo-EM images (micrographs) for tasks such as particle picking or pose estimation (Vulović *et al.* 2013, Bepler *et al.* 2020, Zhang *et al.* 2020, Himes and Grigorieff 2021, Madsen and Susi 2021, Zhang *et al.* 2024). These methods model the physics of electron scattering and the imaging process to produce authentic 2D micrographs. In contrast, our work focuses on synthesizing 3D cryo-EM density maps from atomic models, serving as volumetric representations of molecular structures that are directly used in downstream tasks such as rigid-body fitting and particle picking. While 2D methods aim to simulate the imaging process, our 3D simulation is designed to mimic the structural characteristics of experimental maps. These two directions are complementary. 2D simulations improve image-level analysis, whereas 3D simulations enhance volumetric representations. In addition, an interesting future direction would be to systematically evaluate how both 2D and 3D simulation strategies contribute to improving particle picking.

Overall, by employing *struc2mapGAN*, structural biologists can synthesize improved experimental-like cryo-EM density maps in a timely manner, which can then be used for various applications, such as guiding the particle-picking process before reconstruction of experimental maps and assisting rigid-body fitting with use of simulated maps. Furthermore, machine learning scientists can use *struc2mapGAN* to generate numerous experimental-like density maps as targets, enabling the retraining or fine-tuning of the existing models that were originally trained with simulation-based maps, thereby improving the model performance. Although these generated maps can be useful in early-stage analysis or as supplementary inputs, caution must be exercised when using them for validation, particularly when the underlying atomic models are predicted by AlphaFold or derived from low-resolution data.

While our results demonstrate superior performance in generating improved density maps, it is not possible to relate these maps to an exact resolution value. In principle, it would be feasible to precisely modulate resolutions by integrating a resolution-conditioned modulation mechanism into the generator. This could involve conditioning the network on a continuous resolution parameter via learnable embedding layers or feature-wise linear modulation (FiLM) (Perez *et al.* 2018), allowing explicit control over output resolution. Another promising direction would be to incorporate local resolution information during both training and evaluation. Currently, our model assumes uniform resolution across all voxels. However, in practice, experimental maps often exhibit spatially varying resolution. Future extensions of *struc2mapGAN* could integrate voxel-wise weighting schemes based on local resolution estimates, allowing the model to focus more on high-confidence regions. Similarly, evaluation metrics like correlation and SSIM could be adapted to downweight low-resolution regions, potentially yielding a more biologically meaningful assessment of model performance. Additionally, developing SSE-specific quantitative evaluation metrics would enable more targeted assessment of structural features but remains challenging due to the lack of suitable tools. Furthermore, with advancements in other generative models (Vaswani *et al.* 2017, Ho *et al.* 2020), integrating attention modules into the generator of *struc2mapGAN* could enhance capturing detailed structural information from maps. It would also be interesting to utilize diffusion models for map generation. These will be left for our future work.

Interestingly, our method can also be employed to generate reliable density maps from structures predicted by AlphaFold (Jumper *et al.* 2021) and other de novo sequence-to-structure generative methods (Baek *et al.* 2021, Lin *et al.* 2023), serving as templates to guide and expedite the particle-picking process. For instance, given an AlphaFold-predicted structure, practitioners can generate a 3D density map from its atomic model and extract 2D templates by slicing through the volume. These templates can then be used by particle-picking software to more accurately identify protein particles out of micrographs. While Gaussian blobs can also guide particle picking, template-based approaches are generally more effective, as they are theoretically closer to the actual particles (Sigworth 2016, Cheng 2018). Moreover, these templates have the potential for template matching in cryo-electron tomography (Böhm *et al.* 2000), where consistent SSE representation can improve particle identification. Exploring these applications will also be the focus of our future work.

## Supplementary Material

vbaf179_Supplementary_Data

## Data Availability

The source code of *struc2mapGAN* is available on GitHub at https://github.com/chenwei-zhang/struc2mapGAN. The datasets were derived from sources in the public domain: the EMDB databank [https://www.ebi.ac.uk/emdb/] and PDB databank [https://www.rcsb.org/].
