# Supplementary material for: Struc2mapGAN: improving synthetic cryogenic electron microscopy density maps with generative adversarial networks
Source: Bioinform Adv. 2025 Aug 4;5(1):vbaf179. doi: 10.1093/bioadv/vbaf179 (PMC12360846; doi:10.1093/bioadv/vbaf179)
Supplement: vbaf179_Supplementary_Data [file vbaf179_supplementary_data.zip › Struc2mapGAN_final_SI.pdf]

Supplementary Materials

Struc2mapGAN: improving synthetic cryo-EM density maps with generative adversarial networks

The Supplementary Materials contain:

- Supplementary Table S1
- Supplementary Table S2
- Supplementary Figure S1
- Supplementary Files

Supplementary Table S1

Table S1. List of all EMDB/PDB examples in training and validation sets. Examples in bold are used as the validation set.

EMDB ID	PDB ID	Res. (Å)	EMDB ID	PDB ID	Res. (Å)	EMDB ID	PDB ID	Res. (Å)
26916	7UZQ	2.17	26617	7UN9	3.3	0310	6HZ4	3.6
26917	7UZS	2.2	34963	8HR8	3.3	6324	3JA7	3.6
26886	7UZE	2.4	0089	6GYB	3.3	9105	6ME0	3.6
26974	8CSX	2.4	0690	6J6K	3.3	9111	6MG8	3.6
26948	7V0Q	2.5	3297	5FTL	3.3	30346	7CFS	3.6
26949	7V0S	2.5	4571	6QK7	3.3	34738	8HGG	3.64
14774	7ZL1	2.5	6239	3J9D	3.3	15635	8AT6	3.7
15960	8BC2	2.6	6240	3J9E	3.3	0609	6O2N	3.7
33233	7XJP	2.71	6630	3JCZ	3.3	7041	6B3Q	3.7
27937	8E78	2.77	6932	5ZJI	3.3	8946	6E0F	3.7
27945	8E8O	2.77	6975	5ZX5	3.3	10099	6S59	3.7
26732	7USC	3	9390	6NJO	3.3	10399	6T8B	3.7
26733	7USD	3	10037	6RWB	3.3	20754	6UEN	3.7
26734	7USE	3	10836	6YLE	3.3	20857	6UR8	3.7
0785	6KZ4	3	21863	6WPK	3.3	0379	6N9U	3.7
9322	6N24	3	7006	6AUI	3.3	0688	6J6I	3.7
20442	6PPL	3	9898	6JZO	3.3	4650	6QVE	3.7
21149	6VCD	3	20583	6TYI	3.3	20651	6U5O	3.7
21366	6VRB	3	20968	6V03	3.3	30238	7BXU	3.7
30305	7C8D	3	0327	6I1Y	3.4	27645	8DQ0	3.74
30535	7D0I	3	0923	6LMX	3.4	28080	8EFD	3.8
6714	5XB1	3	0959	6LRR	3.4	28081	8EFE	3.8
21604	6WCA	3	9116	6MHQ	3.4	0935	6LO8	3.8
26838	7UWQ	3.05	9398	6NM9	3.4	7075	6BBJ	3.8
26978	8CT2	3.1	9887	6KUJ	3.4	9971	6KFF	3.8
0590	6O1K	3.1	9905	6K15	3.4	10401	6T8H	3.8
0784	6KZ3	3.1	9954	6KA4	3.4	20700	6U9H	3.8
9253	6MUR	3.1	10208	6SI8	3.4	20471	6PTJ	3.8
10539	6TNY	3.1	10232	6SKZ	3.4	0706	6KLC	3.9
22876	7KHA	3.1	21913	6WUH	3.4	7959	6DLZ	3.9
30016	6LYG	3.1	10206	6SI7	3.4	8185	5JZH	3.9
3999	6EZJ	3.1	33621	7Y5N	3.45	10893	6YS8	3.9
14716	7ZH0	3.2	26841	7UWS	3.47	4749	6R81	3.9
0936	6LOD	3.2	15673	8AUR	3.47	4917	6RLA	3.9
4789	6RB9	3.2	34430	8H1P	3.48	**33719**	**7YAT**	**2.2**
4907	6RKD	3.2	28666	8EY2	3.5	**26973**	**8CSW**	**2.5**
9653	6IFK	3.2	4581	6QLF	3.5	**15361**	**8ADE**	**2.78**
20650	6U5N	3.2	8750	5W0S	3.5	**4798**	**6RBG**	**3**
21146	6VBW	3.2	9104	6MDR	3.5	**6941**	**5ZR1**	**3**
21586	6WB8	3.2	9187	6MP6	3.5	**15646**	**8ATD**	**3.1**
30004	6LX3	3.2	10528	6TMV	3.5	**4890**	**6RIE**	**3.1**
30021	6LZ1	3.2	10573	6TT7	3.5	**15540**	**8ANE**	**3.2**
30022	6LZ3	3.2	20446	6PPR	3.5	**6777**	**5XWY**	**3.2**
30334	7CAL	3.2	20498	6PW4	3.5	**0136**	**6H3N**	**3.3**
0408	6NBD	3.2	20767	6UH5	3.5	**8795**	**5WC3**	**3.5**
5995	3J7H	3.2	22042	6X4S	3.5	**0921**	**6LMV**	**3.6**
20236	6P25	3.2	4746	6R7X	3.5	**14725**	**7ZH6**	**3.67**
20333	6PEV	3.2	21307	6VP9	3.5	**4146**	**5M32**	**3.8**
20334	6PEW	3.2	34679	8HDS	3.57	**30005**	**6LXD**	**3.9**
26616	7UN8	3.3	14873	7ZQP	3.6			

Supplementary Table S2

Table S2. List of all EMDB/PDB examples in the test set.

EMDB ID	PDB ID	Res. (Å)	EMDB ID	PDB ID	Res. (Å)	EMDB ID	PDB ID	Res. (Å)
9590	6ACF	3	30180	7BTO	4	3602	5N8Y	4.7
22414	7JPK	3	6425	3JD6	4.1	6862	5YZ0	4.7
0199	6HCY	3.1	3835	5ONV	4.1	3439	5G5L	4.8
0927	6LN8	3.1	6746	5ZBO	4.1	6875	5Z1F	4.8
9321	6N23	3.1	6987	6A69	4.1	8665	5VFR	4.9
11093	6Z6G	3.1	7018	6AYE	4.1	8954	6E15	5.1
22829	7KDT	3.1	3866	6EGX	4.1	7322	6C05	5.2
0843	6L7E	3.2	4241	6FE8	4.1	3491	5MDX	5.3
9213	6MRU	3.2	4286	6FO0	4.1	9537	5GRS	5.4
0636	6O6R	3.2	4390	6GDG	4.1	4342	6G2D	5.4
20042	6OF4	3.2	9832	6JI1	4.1	8436	5TQW	5.6
10495	6TG9	3.2	0502	6NT5	4.1	10351	6SZA	6
10575	6TTF	3.2	5155	3IYJ	4.2	3885	6EL1	6.1
21481	6VZ1	3.2	3237	5FN2	4.2	8470	5TWV	6.3
10847	6YMX	3.2	3366	5G06	4.2	0608	6O2M	6.3
11488	6ZWM	3.2	4037	5LCW	4.2	1874	2Y9J	6.4
22359	7JK2	3.2	4112	5LVC	4.2	8230	5KBT	6.4
9361	6NF6	3.3	6734	5XMK	4.2	3636	5NG5	6.5
21458	6VYF	3.3	6859	5YYS	4.2	7065	6B7Y	6.5
22131	6XD3	3.3	6940	5ZQZ	4.2	5245	3IZI	6.7
30165	7BSS	3.3	4173	6F2D	4.2	5100	3IXV	6.8
9906	6K1H	3.5	0311	6HZ5	4.2	3761	5O8O	6.8
10213	6SJ7	3.5	20695	6U9E	4.2	6284	3J9T	6.9
20993	6V0C	3.5	8398	5TCP	4.3	3436	5G4F	7
21436	6VXF	3.5	6952	5ZSU	4.3	8097	5IOU	7
4339	6G1K	3.6	7476	6CHS	4.3	8187	5JZT	7.4
0775	6KSW	3.6	7793	6D3R	4.3	7461	6CE7	7.4
0836	6L53	3.6	8919	6DVW	4.3	8685	5VHW	7.8
30071	6M39	3.6	9214	6MRW	4.3	9949	6K9K	7.8
0043	6GOV	3.7	20524	6PYH	4.3	3186	5FJ6	7.9
0257	6HRA	3.7	10273	6SOF	4.3			
0567	6O0H	3.7	10549	6TQE	4.3			
10092	6S3K	3.7	30358	7CGN	4.3			
10214	6SJB	3.7	2364	4BTG	4.4			
21012	6V1I	3.8	6668	5H64	4.4			
10617	6XT9	3.8	8751	5W1R	4.4			
9626	6AHR	3.9	6911	5ZBG	4.4			
0071	6GVE	3.9	7967	6DMW	4.4			
9380	6NIL	3.9	0287	6HV8	4.4			
4537	6QEL	3.9	9870	6JPQ	4.4			
20708	6UAN	3.9	9915	6K4M	4.5			
7118	6BO4	4	0946	6LQI	4.5			
7464	6CES	4	0967	6LT4	4.5			
3984	6EZ8	4	30041	6M1D	4.5			
0088	6GY6	4	5917	4PT2	4.6			
0258	6HRB	4	3776	5OFO	4.6			
9883	6JT0	4	9577	6KV5	4.6			
9118	6MHU	4	22216	6XJX	4.6			
20265	6P6W	4	6535	3JC5	4.7			
20501	6PW9	4	2788	4V1W	4.7			

Supplementary Figure S1

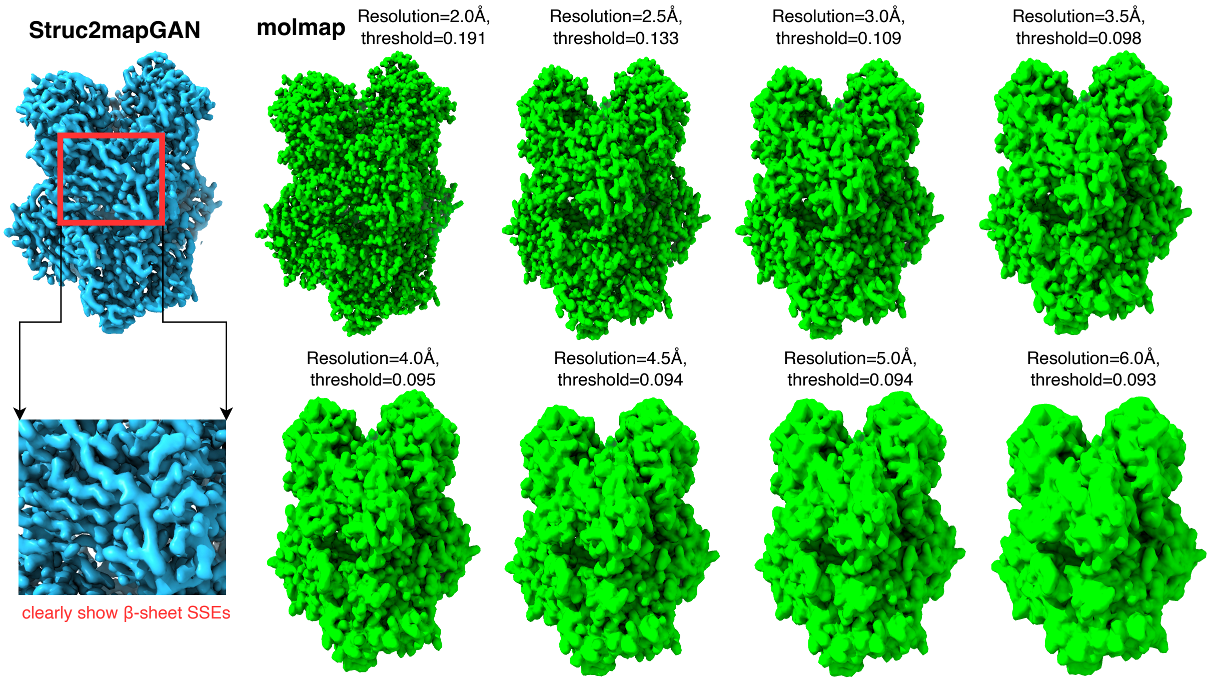

Figure S1. Struc2mapGAN-generated map is shown in blue, and molmap-simulated maps are shown in green, with varying resolution and threshold. The structure visualized here is Truncated HIV-1 Vif/CBFbeta/A3F complex (EMDB ID: 9380; PDB ID: 6NIL; reported resolution: 3.9 Å).

Supplementary Files

All benchmarking and loss results can be accessed at the Supplementary Files:

SI_benchmark_data.csv.
SI_loss_data.csv.
